# Renal Abscess: A Rare Complication of Paratyphoid Fever in an Immunocompetent Traveler from Southeast Asia

**DOI:** 10.4269/ajtmh.21-0368

**Published:** 2021-06-21

**Authors:** Lorenz Schubert, Selma Tobudic, Stefan Winkler

**Affiliations:** Division of Infectious Diseases and Tropical Medicine, Department of Medicine I, Medical University Vienna, Vienna, Austria

A 34-year-old healthy man was admitted to our ward with a 2-day history of severe back pain, malaise, and fever up to 40.5°C after a 2-week backpacking trip to Vietnam, Cambodia, and Thailand. He did not recall any difficulties until 2 days before his return home, when he experienced fever and mild diarrhea after food intake at a local vendor. These difficulties ceased over the course of the following week.

**Figure 1.**
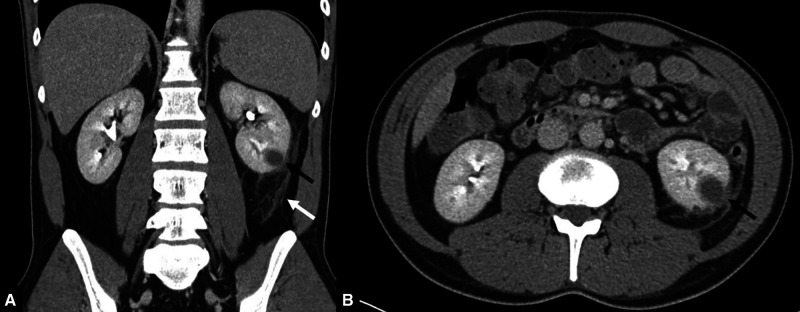
(**A**) Computed tomography imaging after intravenous contrast media injection with multiplanar reconstruction in the coronal orientation at the level of the left kidney shows an abscess (black arrow). This orientation illustrates stranding of the perirenal fatty tissue and thickened Gerota fascia caused by infection (white arrow). (**B**) Computed tomography imaging after intravenous contrast media injection with a slice in the axial orientation at the level of the lower aspect of the left kidney shows a round, hypodense, focal renal mass with irregular margins in the renal parenchyma indicating a renal abscess (black arrow).

On admission, physical examination revealed back pain triggered by percussion of the left kidney; however, no rashes, organomegaly, or confusion was observed. Laboratory test results revealed a normal white blood count (7.6 g/L; normal range, 4–10 g/L), absolute eosinopenia level (0 g/L; normal range, 0–0.4 g/L), elevated C-reactive protein level (11.65 mg/dL; normal range, < 0.5), and elevated lactate dehydrogenase level (435 U/L; normal range, < 250 U/L). Hemoglobin (13.5 g/dL; normal range, 13.5–18 g/dL), platelets (154 g/L; normal range, 150–350 g/L), alkaline phosphatase (44 U/L; normal range, 40–130 U/L), alanine transaminase (30 U/L; normal range, < 50 U/L), aspartate transaminase (42 U/L; normal range, < 50 U/L), and creatinine (1.11 mg/dL; normal range, 0.7–1.2 mg/dL) were within the normal ranges. A computed tomography scan performed to rule out urolithiasis demonstrated pyelonephritis with a concomitant renal abscess and slight splenomegaly (Figure 1A and B).

Calculated antibiotic treatment with cefotaxime 9 g/d was initiated. The next day, two blood culture tests identified *Salmonella enterica serovar Paratyphi A* (*S. paratyphi A*) sensitive to ampicillin, aztreonam, trimethoprim, and levofloxacin but resistant to ciprofloxacin. Interestingly, he did not report receiving a typhoid vaccination. Urine culture and stool culture results remained negative. Abdominal ultrasounds performed after 5 and 10 days revealed no increase in the abscess size. To achieve sufficient antimicrobial blood concentrations after discharge, therapy was adapted to ambulant parenteral antimicrobial therapy with ceftriaxone 4 g/d on day 9. Therapy was continued for a total of 3 weeks. No signs of recurrent infection were reported within 2 months after treatment.

*S. paratyphi* causes paratyphoid fever; furthermore, it is a significant cause of enteric fever in Asia.^1^ In Europe, most cases (97.8%) are associated with travel, and complications are rare because early access to medical care is feasible.^2^ To our knowledge, this is the second documented case of renal abscess caused by *S. paratyphi A* worldwide, and it is the first reported case observed in a returning traveler.^3^ This case report should raise awareness of the unusual clinical manifestations in patients with enteric fever and the increasing number of suppurative *Salmonella* infections.^4^^–^^6^
